# Occupational methanol toxicity: a case report study

**DOI:** 10.3389/fmed.2025.1545428

**Published:** 2025-04-30

**Authors:** Hamoud M. Alrougi, Rayyan Saqah, Imad Khojah, Samar A. Amer

**Affiliations:** ^1^General Directorate of Environmental Health, Ministry of Health, Riyadh, Saudi Arabia; ^2^Department of Emergency Medicine, King Abdulaziz University Hospital, Jeddah, Saudi Arabia; ^3^Department of Public Health and Community Medicine, Faculty of Medicine, Zagazig University, Zagazig, Egypt; ^4^Public Health Agency, Ministry of Health, Riyadh, Saudi Arabia

**Keywords:** methanol toxicity, inhalation, vision loss, metabolic alkalosis, optic neuropathy

## Abstract

This case report describes a case of methanol occupational toxicity in a 44 year-old male ship worker in Jeddah, Saudi Arabia, on 27 August 2024. The patient suddenly lost sight in both eyes and had an unusual acid-base disturbance that was marked by respiratory alkalosis (pH 7.607). This is different from the acidosis that is usually seen in methanol poisoning. Despite aggressive treatment with FOMEPIZOLE, the methanol antidote, and four hemodialysis sessions, the patient’s visual prognosis remained poor. This case highlights the potentially devastating consequences of inhalational and contact methanol poisoning and the importance of prompt recognition and treatment, even in the absence of significant neuroimaging findings. It also underscores the need for proper use of personal protective equipment (PPE) in the workplace.

## 1 Introduction

Methanol is a toxic alcohol commonly found in industrial solvents that can cause serious morbidity and mortality if ingested, inhaled, or absorbed through the skin (1, 2). While oral ingestion is the most common route of severe methanol poisoning, occupational settings increasingly recognize inhalational exposures (3, 4). Formic acid, which has a strong affinity for the optic nerve and disrupts mitochondrial function, metabolizes methanol and leads to visual impairment (5–7).

Vision problems, metabolic acidosis with a high anion gap, changes in mental state, and sometimes neuroimaging abnormalities are all signs of methanol toxicity (8, 9). The severity of poisoning can vary depending on the route and duration of exposure (10). Prompt recognition and treatment are crucial to prevent permanent sequelae (11, 12).

## 2 Case presentation

On 29 August 2024, on arrival at the emergency department, a 44 year-old male seaman, with no prior medical history, visited a hospital in Jeddah, Saudi Arabia. He reported that while working 24 h earlier, he began to experience blurred vision, which gradually increased until he experienced complete, painless, bilateral vision loss, just a few hours after undergoing a methanol wall wash test in an enclosed area.

The exposure history: The exposure occurred on a commercial ship that was loading chemical carbons. The exposure occurred after opening a bottle of 99% concentrated methanol, primarily for a methanol wall wash test. The exposure started at 7:00 pm on 27 August 2024. The exposure route involved dermal contact (arms and face) and inhalation (3 s). The first aid management at the time of exposure involved washing his arms and face and rinsing his eyes under running water for 2 min.

During the general examination: the patient was conscious, following simple instructions, slightly confused, and had a blood pressure of 150/90. During the local examination, the patient’s chest showed equal ventilation. During the eye examination, the patient displayed fixed, dilated pupils and a visual acuity of bare light perception in both eyes. The fundus exam, performed with an indirect ophthalmoscope, revealed bilateral mild disk edema in both eyes, along with nerve fiber layer edema and whitish edema along the arcades.

The laboratory finding: The following results were normal: HB, PLT, CA, MG, LFTS, albumin, TREP, CK-MB, lactate, RFTS, D-DIMER, coagulation profile, LDH, triglyceride, cholesterol, and CRP. While the pH is 7.5, HCO3 is 24. The first tests in the lab showed that the person’s respiratory system had respiratory alkalosis (pH 7.607), low pCO2 (12.5 mmHg), and low HCO2 (19.8 mEq/L). Sequential arterial blood gas (ABG) measurements throughout his hospital stay demonstrated persistent alkalotic pH values, ranging from 7.397 to 7.607. This is detailed in (Tables 1–3).

**TABLE 1:** The biochemistry subsequent reading of methanol exposure case.

The biochemistry variables	The subsequent readings
Sodium (na)	133, 141 > 138 > 137 > 139 > 139 > 137
Potassium (k)	2.9, 3.3, 4.2 > 4.1 > 4.4 > 4.3 > 4.2 > 4.6
Hco3	15 > 25 > 22 > 28 > > 25
Lipase	255, 445 > 395 > 232 > 316 > 424 > 300
Phosphorus (po4(	0.6, 0.2, 0.7, 1 > 0.8 > 1.2
Amylase	134 > 119 > 149 > 147
Ca	2.06 > 2.06 > 2.11 > 2.22
Serum osmolality	304, 279 mmol/kg H_2_O
Magnesium	0.8
Total bilirubin	21.1
Triglycerides	1.02 mmol/L (normal)
Troponin mag	3.59 pg/ml

**TABLE 2:** The hematological finding of methanol exposure case.

The hematological variables	The subsequent readings
White blood cells (WBC)	19.44 > 13.3 > 15.5 > 19.4 > 20.4 > 19.2 × 1,000/μL
HB	14.1 g/dL (normal)
Eosinophils	00% (normal)
D-dimer	2: 0.29 mg/L (normal)
INR2	1.06 (normal)
Lymphocytes	05% (normal)
MCHC	35.3 gm/dL
Monocytes	02% (normal)
MCH	28.3 pg (normal)
MCV	80.2 fL (normal)
Neutrophil segmented	3% (normal)
HCT	40.0% (normal)
Platelet	392 × 1,000/μL (normal)
PT2	12.1 (normal)
PTT2	25.9 (normal)
RBC	4.99 × million/μL (normal)
RDW	12.9% (normal)

**TABLE 3:** The serological and hormonal of methanol exposure case.

The serological and hormonal variables	The subsequent readings
CRP3	0.5
4 TSH ALL	0.31 μIU/mL

**FIGURE 1:**
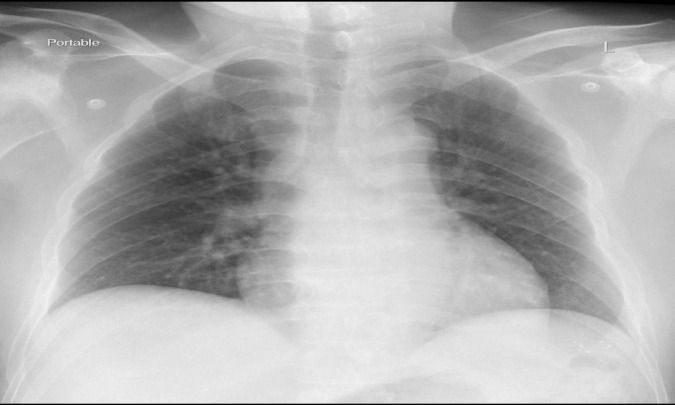
The plain chest X ray among the case exposed to occupational methanol inhalational.

Imaging studies were performed to assess the effect or the potential complications. The chest X-ray revealed bilateral peribronchial cuffing is seen along with prominent pulmonary vasculature, but no well-formed airspace opacity (Figure 1).

While the initial non-contrast, and contract CT brain was unremarkable, showing no cerebral parenchymal abnormalities or intracranial hemorrhage.

**FIGURE 2:**
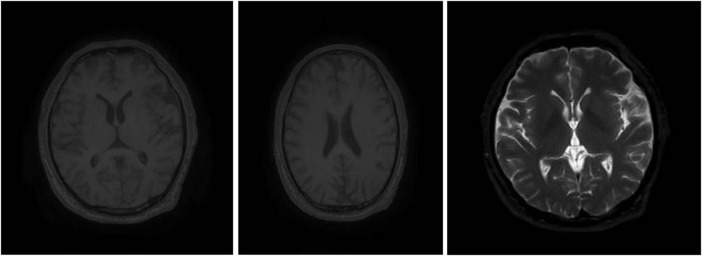
The MRI brain case exposed to occupational methanol inhalation.

The MRI brain preserved the differentiation between gray and white matter, revealing normal preservation of the gray-white matter difference, intracranial vascular signal voids, supratentorial and infratentorial brain parenchyma. The orbits and bones are unremarkable. There was no evidence of acute infarction, hemorrhage, hydrocephalus, herniation or space-occupying lesions. The only abnormal finding noted was linear ethmoidal and maxillary sinus mucosal thickening, which was likely incidental. MRI revealed no optic nerve abnormalities as in Figure 2.

**FIGURE 3:**
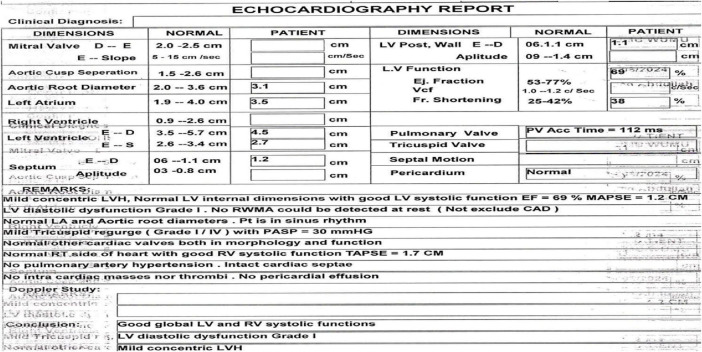
The Echocardiography report case exposed to occupational methanol inhalation.

A heart scan using echocardiography showed mild concentric left ventricular hypertrophy. The systolic function was normal (ejection fraction of 69%), but the diastolic function was Grade I as in Figure 3.

The differential diagnosis (DD) for rapid or acute vision loss may be chemical eye burns, uveitis, retinitis, keratitis, optic nerve damage, neurotoxicity, encephalitis, and meningitis. After imagining the linear ethmoidal and maxillary sinus mucosal thickening, we can think of diagnosing acute ethmoiditis, fungal, bacterial, or viral infections. Chronic ethmoiditis: These include sinusitis, allergic rhinitis, sarcoidosis, and Wegener’s granulomatosis, with ethmoid sinus tumors as DD.

But after considering the history, examination, and investigations, the initial diagnosis was methanol toxicity and complete blindness. The management approach involved admitting the patient to the intensive care unit (ICU) and intubating them for airway protection. Careful electrolyte management was instituted as recurrent hypokalemia and hypophosphatemia. The patient got 13 doses of fomepizole, which is an alcohol dehydrogenase inhibitor, methylprednisolone 1,000 mg IV every day for 3 days, thiamine 500 mg IV every day for 3 days, folate 5 mg every day, and extra electrolytes.

Cobal 500 mcg bd; Ponizex 40 mg i.v. od; Inhixa 40 mg c/c od; Folic acid 5 mg od; Folicum 5 mg od; Biosoft Plus eye drops 1 drop q2h; Refresh eye drops 14 mg q2h; Solu-Medrol 80 mg i.v. od, and 70 mg i.v. od for 5 days; Pyridoxine 40 mg po od; Thiamine 200 mg i.v. od; Blum-D 50,000 IU o/w.

Regarding the operations or procedures, the patient underwent catheterization, cannulation of other VEIN, four hemodialysis sessions, a referral to a nephrologist, and dialysis. The total ICU length of stay was 5 days.

During the patient’s stay in the hospital, repeated ABG tests showed persistent respiratory alkalosis. By 1 September 2024, the patient’s ABG showed a pH of 7.468, low pCO2, and a base excess of 5.1 mEq/L, confirming ongoing respiratory alkalosis.

On the third day of hospitalization, the patient underwent extubation, yet he still lacked light perception in both eyes. A fundoscopic examination showed optic disks that were slightly swollen.

At the time of discharge on Hospital Day 11 (8 September 2024), the final diagnosis was methanol toxicity and binocular blindness. We started him on an oral prednisone taper (5 mg Table 30’s “regular” dose = 70 mg once daily for 1 week); Cobal 500 mcg Table 30’s “regular” dose = 500 mcg twice daily for 1 week; Pan Tomax 40 mg Table 30’s “regular” dose = 40 mg twice daily for 1 week; Blum-d 50,000 IU Table 20’s “regular” dose = 50,000 IU once a week. The patient was discharged with instructions for outpatient nephrology and ophthalmology follow-up. Unfortunately, due to deportation to his home country, no follow-up information was available to assess his long-term outcomes. The exposure route in this case—inhalation combined with dermal contact—is less common than ingestion but increasingly recognized in occupational settings (3).

## 3 Discussion

Since the late 1800s, methanol has become a medical problem because new manufacturing techniques have made it more available. This type of exposure—inhalation combined with skin contact—is less common than ingestion but is becoming more recognized in the workplace (3). In addition to that, skin exposure or fume inhalation can rarely lead to toxicity (13).

So, this case report shows what happens when you breathe in windshield washer fluid for 3 s or touch methanol that is toxic for one minute and how hard it is to deal with the acid-based problems that come with it. According to Beauchamp and Valento (14), most methanol poisonings occur due to accidents or suicide attempts involving the consumption of windshield washer fluid. Moreover, the patient’s lack of adherence to personal protective equipment (PPE) protocols likely contributed to the severity of his poisoning. This underscores the critical importance of stringent workplace safety measures and proper PPE use to prevent exposure (10).

The patient sought medical advice or visited the hospital 27 h after the exposure, when the second wave of symptoms appeared. Following inhalation, methanol produces a narcotic effect, leading to central nervous system depression (15). The individual then becomes asymptomatic for 10–15 h (16). After that, the second set of symptoms, which include blurred vision or complete vision loss, appear 10–30 h after exposure—a rare but significant consequence.

Physical examination may reveal dilated pupils, optic disk hyperemia, and retinal edema. According to Permpalung et al. (17) and Jafarizadeh et al. (18). On the third day of hospitalization, the patient still lacked light perception in both eyes. A fundoscopic examination showed optic disks that were slightly swollen, which is a sign of toxic optic neuropathy (19, 20). While MRI revealed no optic nerve abnormalities, in contrast to some reported cases (1, 21). The onset of symptoms was explained by Wood and Buller who found that neurotoxic symptoms including (acquired sudden blindness or near blindness, with widely dilated, reactionless pupils, sluggish breathing, weak pulse, diaphoresis, or dementia) often appeared 24 h or more after exposure (22).

The causes of vision loss that result from occupational exposure to windshield washer fluid inhalation (3 s) and methanol contact toxicity (1 min) that may be attributed to methanol include conditions like optic neuropathy, corneal ulcers and scarring, neurotoxicity, inflammation of the eye, damage to the optic nerve, brain swelling, and damage to the visual pathway. Furthermore, additional potential causes of vision loss because of methanol exposure include these: Acute angle- Closure glaucoma- Optic neuritis- Retinal detachment - Cataract (23, 24, 25).

Based on the reported clinical symptoms per examination at the hospital, it is considered moderate methanol poisoning according to Wood and Buller, who categorized the poisoning severity into three levels: (1). Mild intoxication with dizziness, nausea, and mild GI upset, which cleared in a few days but sometimes caused vision impairment. (2). Moderate poisoning, with conspicuous dizziness, nausea, vomiting, diarrhea, and headache coupled with dimness of vision, often escalating to total blindness. (3). Severe poisoning, with an overwhelming prostration, which terminates in coma and death.

During the initial laboratory investigation, it was shown that the person’s respiratory system had respiratory alkalosis (pH 7.607), low pCO2 (12.5 mmHg), and low HCO2 (19.8 mEq/L). This was different from the typical metabolic acidosis seen in people who have been poisoned by methanol. Methanol became increasingly recognized as a cause of death, metabolic acidosis, and permanent blindness by the early 20th century.

Therefore, instituted careful electrolyte management due to the unusual presentation of alkalosis rather than the expected acidosis. During the patient’s stay in the hospital, repeated ABG tests showed persistent respiratory alkalosis. Most likely, methanol or its metabolites directly stimulated respiratory centers, causing hyperventilation. By 1 September 2024, the patient’s ABG showed a pH of 7.468, low pCO2, and a base excess of 5.1 mEq/L, confirming ongoing respiratory alkalosis. This management approach aligns with current treatment recommendations for severe methanol poisoning (11, 12, 26).

The patient in this case report presented with respiratory alkalosis (pH 7.607) as the primary acid-base imbalance. According to McCurdy (27), patients who are having primary ventilatory problems often have respiratory alkalosis or acidosis. This is different from the metabolic acidosis that is common with methanol poisoning and has a big impact on how they interact with their breathing problem. This unusual presentation can be attributed to central respiratory stimulation, a less recognized feature of methanol toxicity that may occur due to the direct effects of methanol or its metabolites on the respiratory centers of the brain (11, 28).

The patient in this case received treatment with Fomepizole selectively inhibits methanol metabolism, an alcohol dehydrogenase inhibitor, was administered as an antidote for methanol toxicity, and several sessions of hemodialysis, which remains the most effective method for eliminating methanol and addressing issues with acid-base balance. Folate supplementation was also administered to enhance the metabolism of formate, a key toxic metabolite (11). Despite the use of steroids to reduce optic nerve inflammation, their effectiveness in enhancing outcomes is still unknown (29).

Despite aggressive treatment, the patient experienced severe and permanent visual deficits, a common outcome in severe methanol toxicity cases in agreement with other studies (30, 31). Early recognition and intervention remain crucial for improving prognosis (32, 33).

At discharge, nephrologist follow-up is required. This is due to the sluggish excretion of methanol (t 1/2 = 24 h), which primarily manifests as formic acid in urine. Peak serum concentrations occur within 30–90 min. Methanol transports poorly to fatty tissues and does not bind plasma protein. Formic acid excretion from methanol is mostly urine. At high concentrations, methanol elimination reaches saturation and zero-order at approximately 85 mg/L, resulting in a 50% efficiency in ethanol removal. Formic acid excretion may peak 2 or 3 days after consumption. The lung and kidneys eliminate 2% of 50 mg/kg methanol unaltered. Urine methanol may be 20%–30% higher than blood [Geneva: (34)].

Therefore, we must design occupational safety and eye health programs to protect workers’ vision. The International Labor Organization estimates that 3.5 million industrial eye injuries occur annually, impacting over 13 million workers (35). Occupational safety and eye health programs should focus on keeping people from being exposed to dangers at work, protecting eye health, and making sure that people who naturally lose their sight are covered by insurance. Expanding workplace eye care interventions to include two main interventions is necessary to reduce preventable vision impairment. (1) Making sure that everyone wears the right eye protection, along with (2) controlling the environment at work and encouraging people to follow the best practices for using eye protection (36–40).

The strength of this case report is that it provides a detailed description of a relatively rare occupational exposure to methanol by inhalation and contact, including full clinical, imaging, and laboratory findings as well as management.

One limitation of this report is the absence of long-term follow-up. Shortly after discharge, the patient’s deportation to his home country prevented further evaluation of his visual recovery or delayed complications. This highlights the challenges in ensuring continuity of care for patients exposed to occupational toxins, particularly in transient or migrant worker populations.

## 4 Conclusion

This case illustrates the consequences of inhaling or touching methanol at work, including the potential for permanent and severe vision loss, even with prompt and aggressive treatment. The atypical presentation of respiratory alkalosis in this patient deviates from the classic metabolic acidosis typically associated with methanol poisoning. It shows how acid-base imbalances can change depending on the person. Methanol or its metabolites likely cause hyperventilation by directly stimulating the breathing centers.

The absence of optic nerve abnormalities on neuroimaging, despite profound visual loss, underscores the importance of clinical evaluation in diagnosing and managing methanol toxicity. Early recognition based on clinical findings, rather than reliance on imaging, remains critical for timely intervention.

## 5 Recommendation

• Careful electrolyte management is essential in the management of ethanol toxicity.
• Only well-trained personnel should use concentrated forms of methanol and other toxic agents.
• Researchers should find safer alternatives to such toxic products.
• Preventative measures are paramount, including the strict enforcement of personal protective equipment (PPE) protocols, proper workplace ventilation, and comprehensive safety training for individuals working with methanol. These strategies are essential to reduce the incidence and severity of such toxic exposures.
• Finally, this case highlights the need for ongoing monitoring and long-term follow-up to better understand the spectrum of outcomes associated with inhalational methanol toxicity. Enhanced education and awareness among healthcare providers and occupational safety personnel are crucial to ensure early detection, effective intervention, and prevention of morbidity and mortality associated with methanol exposure in occupational settings.

## Data Availability

The raw data supporting the conclusions of this article will be made available by the authors, without undue reservation.
